# Sustainable Alternatives to the Reduction of Plastic Straws Used with Chilled Equine Semen

**DOI:** 10.3390/ani14233388

**Published:** 2024-11-25

**Authors:** Noelia González, Aroa Peñalosa, Ignacio de Blas, Lydia Gil

**Affiliations:** 1 Department of Animal Pathology, Faculty of Veterinary Sciences, Universidad de Zaragoza, Miguel Servet 177, 50013 Zaragoza, Spain; 728296@unizar.es (A.P.); deblas@unizar.es (I.d.B.); lydiagil@unizar.es (L.G.); 2 Instituto Universitario de Investigación Mixto Agroalimentario de Aragón (IA2), Universidad de Zaragoza, 50013 Zaragoza, Spain

**Keywords:** spermatozoa, preservation, biodegradable

## Abstract

Currently, plastic is used being without any limitations. The livestock sector is no stranger to its use. For example, artificial insemination involves the use of different plastic materials, including semen straws and insemination catheters, which increase the levels of waste in our environment. Finding a sustainable solution to avoid plastic materials is a challenge. The aim of this study was to examine different biodegradable materials as alternatives to conventional syringes used for refrigerated equine semen. The materials tested were bamboo, avocado, grass, paper, Kraft paper, wheat, and rice. Bamboo and avocado were selected to continue testing with the refrigerated semen with a lifespan extended by INRA 96^®^; the rest of the materials did not meet the requirements necessary for preservation. Motility and movement kinetics tests were performed at 24 and 96 h of refrigeration to evaluate semen quality; relative to the control, significantly better results were displayed in regard to the total and progressive motility avocado straws. The kinetic parameters were comparable to the control for both materials and within normal ranges. After performing a microbiological control process for both straws, it was confirmed that there was no bacterial growth in either straw. We can confirm that avocado straws can be used to preserve chilled equine semen, guaranteeing seminal quality while being respectful to the environment.

## Simple Summary

The situation in which our society finds itself regarding the excessive use of plastic materials in all areas is worrying due to the environmental pollution it generates, and the veterinary field is no exception to this situation, since the use of plastic is widespread in daily work. The replacement of plastic materials with more sustainable ones is urgent, and that is why the search for these elements is so important and a starting point for collaboration in the reduction in waste. Artificial insemination is a technique frequently used with equine species and involves the use of plastic materials such as insemination catheters and semen straws. Finding alternative biodegradable materials is the aim of this work, evaluating the use of straws made of bamboo, avocado, grass, paper, Kraft paper, wheat, and rice. The materials that offered the best results as containers for semen samples were bamboo and avocado, the latter being able to maintain the quality parameters of the sperm it contained without the presence of contamination, so we could consider it a viable alternative to the use of conventional plastic straws, thus contributing to a reduction in waste.

## 1. Introduction

Currently, there is great concern about plastic accumulation. In our environment, almost all sectors use common, durable, and difficult-to-degrade plastic in their activities; these plastics are transformed into microplastics, contributing to another serious environmental problem [1,2] that can affect elements of animal health, including the nervous system [3], digestive system [4], and reproductive system [5]. Plastic use is even present in the veterinary world [6]. There are numerous containers that are made with nonbiodegradable plastic, such as syringes, medicine containers, gloves, probes, and the chilled or frozen semen straws used for artificial insemination (AI) in different species; these straws have been utilized since 1964 when Jondet [7] and Cassou [8] started using them with bovine semen to reduce the large volume of glass used for preservation. Glass was the material employed at that time. Even the insemination catheters used in some cases are composed of plastic, and, given that it is a very widespread technique in animal reproduction, the amount of waste generated using this type of material is considerable. The challenge is to find a method for eliminating or reducing this waste. One technique to achieve this goal is to use biodegradable materials in all areas in which conventional plastic can be replaced in order to reduce pollution [9]. Biodegradable plastics are plastics that undergo degradation reactions carried out by microorganisms, such as fungi and bacteria, mainly under certain conditions. These processes take place under soil, water, and composting conditions [10].

Through AI, we found seminal cryopreservation; in fact, AI and seminal cryopreservation are two reproductive techniques that have been advancing together since it is unthinkable to discuss AI without cryopreserved sperm. In both techniques, the use of plastic is present. It is necessary to determine which other types of materials are the most appropriate when in direct contact with semen, and the influences that these materials may have on sperm viability must be evaluated. Sperm cryopreservation is a technique that can affect sperm quality parameters, such as morphology, motility, viability, and DNA integrity through cryoinjury. Therefore, the straw products used in preservation should not cause any damage to the sperm from the materials that they release and that affect them. Likewise, it must be considered that these products can damage the reproductive tract of the mare where the semen is deposited, implying its inflammation, which can diminish the success rate of fertilization. In fact, some plastics may have a negative influence on semen quality [11]. For replacements, there are several feasible options.

Bamboo fiber is obtained from the plant through a chemical or mechanical process. This fiber can be used to make paper, textiles, nanomaterials and other compounds. The high cellulose content, basic mechanical properties (bundles), sufficient specific strength [12], and high percentage of lignin (32%) make bamboo a good option for replacing plastic. Moreover, bamboo is a biodegradable material that can be eliminated by soil burial and by degradation [9].

Another material that can be used as a substitute for plastic is avocado seeds from the waste of companies that make guacamole and avocado oil. Avocado seed residues have been shown to contain 30% starch, which is usable as a bioplastic material [13], and these bioplastics have been shown to exhibit biodegradable properties; moreover, their high antioxidant [14] and antifungal properties [15] are very interesting.

Grass is another packaging option that is considered part of composting and is not harmful to the environment. In the composition of grass, there are cellulose, hemicellulose, lignin, and ash [16].

Paper is a widely used element in everyday life, and it allows us to reduce pollution. A variant, Kraft paper, can be obtained from wood fiber pulp, giving it a resistant quality. While conventional paper is biodegradable, Kraft paper is not considered as such by some scholars [17] since, after 45 days of composting, it only has 36% organic matter relative to the 45% that is needed for such treatment. However, paper biodegradation depends on the composting conditions, since, at 50 °C, it degrades as expected [18].

Another biodegradable material is wheat, which contains gluten, a protein byproduct of starch manufacturing. Given its composition, the material biodegrades after 50 days conventionally or after 36 days under aerobic fermentation conditions [19]. Wheat straw biomass consists mainly of lignocellulosic materials, but the different parts of the plant have distinct variations in their carbohydrate and lignin levels [20].

Finally, rice, which is one of the most produced cereals in the world, contains a large amount of starch and is gluten-free. Straws can be made with rice flour, wheat flour, tapioca starch, and water.

By taking into account the existence of these biodegradable materials, the aim of this work will be to explore and study the use of these materials for sperm chilling in the equine species, replacing the plastic materials used.

## 2. Materials and Methods

### 2.1. Chemicals and Media

Unless otherwise stated, all chemicals were sourced from Sigma-Aldrich Co. (Alcobendas, Madrid, Spain), except for the INRA 96^®^ (Rf. 016441, IMV Technologies; L’Aigle, France) and EquiPlus^®^ (Rf. 13570/0222, Minitube, Tiefenbach, Germany) extenders. For microbiological control, 3 M Petrifilm^®^ Plates kits (3 M, Barcelona, Spain) were used for aerobic (Rf. 6400/6406/6442) and enterobacteria (Rf. 6420/6421) counts.

### 2.2. Sperm Collection

Testicle samples of twenty horses from the municipal slaughterhouse of Zaragoza, Mercazaragoza S.A., without morphological alterations and with an intact epididymis and an intact vas deferens of adequate size were used. Once the testicular sheaths were removed, the epididymis and vas deferens were separated from the rest of the testis. Vas deferens were cannulated with a 25G needle connected to a 10 mL syringe, and retrograde lavage was performed with INRA 96^®^ diluent to obtain the sperm. The samples were placed in a beaker and held at 30 °C until evaluation. Next, 4 µL of the sample (diluted 1:100 with INRA 96^®^) was taken and placed between slides and coverslip to analyze sperm motility using the integrated sperm analysis system ISAS^®^ V1.2 (Projectes I Serveis R + D S.L., Valencia, Spain) to determine the suitability of the sample for the experiment, selecting only samples in which all sperm parameters were in the normal ranges for equine semen: 78% motility, 120 × 10^6^ spermatozoa/mL, and 75% normal morphology. Once the seminal quality was verified, the pooled sample was centrifuged (600× *g*, 10 min) after dilution in INRA 96^®^ (concentration 100 × 10^6^ sp/mL) to eliminate the extender and thus be able to work with the selected extender in each case (INRA 96^®^ for the preservation tests and Equiplus^®^ for the microbiological controls). Five replicates were made with four horses for each one.

### 2.3. Biodegradable Straws and Tested Sealing Systems

The biodegradable straws tested in the study and their trade names are described in Table 1. The control were 20 mL syringes (Braun Injekt^®^ Luer Solo).

The following five sealing systems were evaluated: polyvinyl alcohol (PVA), polystyrene balls (diameter 4, 5 or 6 mm), cork (4, 5 or 6 mm diameter × 8 mm long), rice wax, and beeswax. These sealing systems were tested in all types of straws used in four replicates.

Sealing formats:

− PVA is a powder element that solidifies upon contact with water. To seal, it is necessary to first introduce it into the straw end and then put it in contact with water. In this manner, a solid plug was achieved.
− Polystyrene balls: Despite having different diameters, since the straws used were different, they rarely fitted perfectly; thus, the closure had to be completed using PVA and a polystyrene ball.
− Cork sealing: Stoppers with different diameters were made from a 0.5 mm sheet of cork. Despite the variety of diameters, the use of PVA was necessary to ensure sealing and a perfect fit with the straws.
− Waxes: For their management, it was necessary to soften the samples with heat to be able to manipulate them and give them the appropriate shape so that they would fit into the diameter of the straws. A 0.5 cm-thick film of wax was prepared on a Petri dish and slightly heated so that, when the straw was placed over it, the wax entered it and remained as a plug.

### 2.4. Wettability Tests for Straw Selection

To evaluate the permeabilities of the straws, a wettability test was performed. A water droplet was placed on the surfaces of the different straws, and the formed contact angle was measured from the three-phase boundary where liquid, gas, and solid intersected; we determined if there was a change in them as a consequence of the absorption of the contained liquid. The measurement was performed with an optical tensiometer (ThetaLite, DYNE Testing) on a vibration isolation system (Halcyonics Micro 40).

### 2.5. Sperm Chilling Evaluation

The sperm chilling process was developed with the straws and sealing system selected in the preliminary test: avocado and bamboo straws and cork combined with PVA for sealing.

The extender used was INRA 96^®^ for chilling protocol and Equiplus^®^ for microbiological control. Once the straws were loaded with the semen sample, they were chilled at 5 °C for 96 h, performing seminal quality control at 24 and 96 h. The control was a sample preserved in a plastic syringe.

Total motility and velocity parameters were analyzed through ISAS^®^, with the samples and material being tempered at 37 °C. The parameters established for the analysis were 25 consecutive digitalized images per second, and the particle area was 4–75 μm^2^. With regard to the setting parameters for the sperm, values of VAP < 10 μ/s were considered slow, and those >90 μ/s were considered fast. Spermatozoa with 75% of the straightness index (STR) were designated as progressive motile sperm. The displacement amplitude (ALH) value was 10 μm (minimum number of images calculated). Total and progressive motility data and different motility kinetic parameters were collected: curvilinear velocity (VCL), rectilinear velocity (VSL), average speed (VAP), linearity index (LIN), straightness index (STR), oscillation index (WOB), and the average amplitude of displacement (lateral width of the head: ALH and beat frequency: BCF). For the study of these parameters, three different fields of each sample were randomly captured.

### 2.6. Microbiological Control

Microbiological controls were carried out at 0, 24, and 96 h for both aerobic and anaerobic bacteria. To test avocado and bamboo straws, they were filled with semen extended with EquiPlus^®^, an extender lacking an antibiotic that could control bacterial growth.

Four controls were prepared in Eppendorf^®^ tubes: INRA 96^®^, INRA 96^®^ with semen, EquiPlus^®^, and EquiPlus^®^ with semen.

All samples were prepared as follows: a 1/100 dilution was made in physiological serum, and seeding was carried out with Petrifilm^®^ AC and Petrifilm^®^ EB plates. The samples were held in a 37 °C oven, and the presence of bacteria was later evaluated.

### 2.7. Statistical Analysis

The variables collected were described by calculating the standard deviation. The effects of time and types of straw on the different variables analyzed were evaluated using a generalized linear model (GLM) of repeated measures assuming an alpha error of 0.05. A pairwise comparison of the types of straw at each time point was carried out with paired data using a Wilcoxon test. Both analyses were performed with IBM SPSS Statistics 19.0 for Windows.

## 3. Results

### 3.1. Straw Storage Capacity

Due to the fragility of wheat and grass straws, these specimens were the first to be discarded despite not having any negative effect on sperm quality.

**Figure 1:**
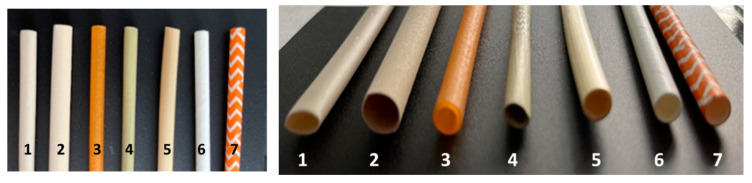
Images of the avocado (1), bamboo (2), rice (3), wheat (4), grass (5), paper (6), and Kraft paper (7) straws.

After assessing the storage capacity, three replicates were made, with five straws of each type (Figure 1). To assess resistance to humidity, a wettability test was performed (Table 2), and it was important to determine whether straws were able to maintain the content.

**Figure 2:**
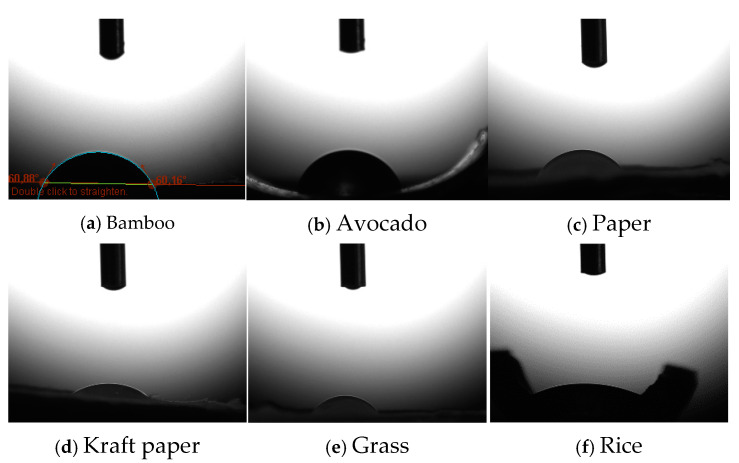
Wettability test to check the permeabilities of tested straws: bamboo, avocado, paper, Kraft paper, grass, and rice.

Bamboo and avocado straws were able to maintain the liquid content at 5 °C for the established time with three sealing systems (Figure 2a,b), maintaining the same angle inclination and volume during the wettability test. The paper, Kraft paper, and wheat straws absorbed the content completely (Figure 2c–e), losing the liquid. The grass straws (Figure 2e) were unable to maintain the content and appeared to be degraded. The rice straws were discarded because they retained the content for only 4 h before disintegration (Figure 2f). Sealing with polystyrene and polyvinyl alcohol (PVA) balls was effective for the bamboo, avocado, and wheat straws both at 24 and 48 h; despite the results, given that the polystyrene balls were not biodegradable, it was better to discard this option from the experiment. Despite the results obtained with stoppers of two wax types during refrigeration, which were possibly effective in retaining the liquid, especially in the avocado and bamboo straws, the system was discarded because of the difficulty of handling. The cork with PVA sealing was effective for avocado and bamboo straws. With these results, we selected bamboo and avocado straws to continue the experiment with seminal samples with the cork sealing system.

### 3.2. Semen Quality During Refrigeration

**Figure 3:**
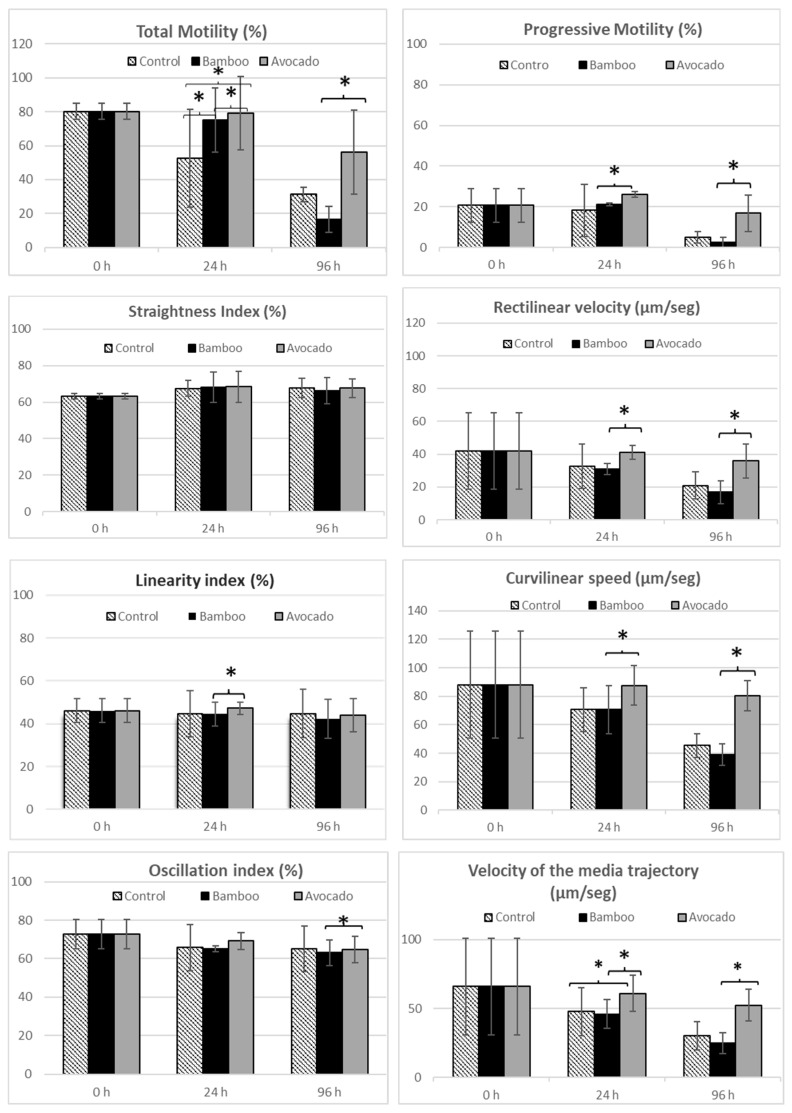
Time evolution for different parameters of motility for the control, bamboo, and avocado straws at 0, 24, and 96 h of refrigeration. The progressive total motility, straightness index, linearity index, and oscillation index values were expressed in percentages. The rectilinear velocity, curvilinear speed, and velocity values of the media trajectory were expressed in µm/seg. Asterisks indicate significant differences (<0.5 according to Wilcoxon test) between the type of straw and time.

During seminal preservation, motility and kinetic parameters were assessed in the control and in two types of the selected straws (Figure 3).

Total motility showed significant differences (*p* < 0.050) regarding the type of straw, presenting the best results for the avocado straw in all the conservation times in which it was contrasted; in fact, at 24 h, the value was much higher than that of the control (79.2 ± 21.77 vs. 52.53 ± 28.74) (Figure 3). For the bamboo straws, at 24 h, the values were similar to those of the avocado and control. Nevertheless, at 96 h, the results from avocado were strikingly higher than those from bamboo (56.27 ± 24.75 vs. 16.57 ± 7.60); however, the difference from the control was not as marked, although it should be considered.

Regarding progressive motility, there were no differences in terms of straws or between the different times, but the results at 24 h were high for avocado, followed by bamboo. At 96 h, avocado performed the best, obtaining striking results.

### 3.3. Linearity Index (LIN), Oscillation Index (WOB), and Straightness Index (STR)

No significant differences (*p* > 0.050) were found between linearity, oscillation, and straightness depending on the material, not even in the time they were stored; this finding indicated that the material used was not relevant based on the conserved form and that any of them could be valid.

### 3.4. Rectilinear Velocity (VSL), Curvilinear Velocity (VCL), and Average Speed (VAP)

Significant differences (*p* < 0.050) in these variables were found with respect to the type of straw (Figure 3). The VSL at 24 h for the avocado straws was higher than that for the control and bamboo (41.05 ± 4.11 vs. 32.65 ± 13.43 and 30.78 ± 3.35, respectively). At 96 h, the same trend was observed (35.79 ± 10.33 vs. 20.8 ± 8.29 and 16.87 ± 7.05). Bamboo had the poorest value. The VCL data showed that, at 24 h, the values of the control and bamboo (70.58 ± 15.33 vs. 70.57 ± 16.81) were very similar and lower than that of avocado (87.57 ± 13.81). At 96 h, the poorest value was bamboo (38.95 ± 7.70), which was followed by the control (45.36 ± 8.52) and surpassed by the value of avocado (80.45 ± 10.57), being the best with a notable difference. For VAP, the same situation was repeated; at 24 h, the avocado value was higher than that of the control and the bamboo (60.99 ± 13.03 vs. 47.63 ± 17.4 and 45.97 ± 10.43), and, at 96 h, the same trend followed (52.35 ± 11.61 vs. 30.24 ± 10.28 and 24.89 ± 7.52).

### 3.5. Bacterial Growth

In the assay designed to check the bacterial growth in the selected straws with equine semen with a lifespan extended by EquiPlus^®^ after storage at 0, 24, and 96 h, no growth was observed in any of the samples at any time.

## 4. Discussion

In this study, we have proposed working with different biodegradable straws made from vegetal resins. Regarding biodegradable materials for reproductive purposes, in the livestock world, an insemination catheter for swine composed of biodegradable materials has been on the market since 2020, the iGreen^®^ ecocatheter (Magapor, Ejea de los Caballeros, Spain); however, we are unaware of research involving other animal species using this type of material for reproductive purposes, including catheters and straws.

It is difficult to find the optimal material; moreover, it must be considered that the straws have to be sealed to retain the semen extended inside them, and finding a sealing system is another challenge.

In our case, not all the straws tested were a good option. After cryopreservation, the paper straws did not present any content; in fact, the wettability test showed that it was not a good option, and sealing was not important because the content could have been embedded in the straw itself and not lost through the stopper. Nevertheless, the beeswax sealing method was discarded, as the results reflected the loss of content, possibly because the sealing, although initially correct, caused the wax plugs to retract when cold, causing the seal to partially shrink. Kraft paper retained the samples for 24 h because it was much less hygroscopic than paper, and its absorptivity was reduced significantly by the impregnation treatment to which it was subjected. Perhaps, if we had used Kraft paper sealed with chitosan, the straws could resisted for 48 h, as this treatment decrease the straw permeability [21] and was not toxic for spermatozoa when used in low concentrations [22], even if it could be beneficial for them because of its antioxidant action [23]. The rice straws disintegrated in 4 h, possibly due to their composition, since they were mostly composed of wheat flour, which is a water-soluble material; therefore, when exposed to the diluent, they disintegrated. Grass straws were not a good option either; they were not capable of maintaining their content, and they were difficult to handle due to their fragility and low resistance to bending, an inherent characteristic of this material, similar to other types of herbs [24], making it an inappropriate material for this use. In addition, we had to address the degradation that these straws could suffer internally due to their exposure to the extender, which could be harmful to the semen due to the substances released if they were straws that endured the entire refrigeration period.

The best results were obtained with bamboo and avocado straws, which retained the samples for 48 h. In the case of bamboo straws, the material with which they were made guaranteed durability: bamboo fibers were very resistant, and, depending on their treatment, they could become resistant and flexible [12]. Additionally, the strength and flexibility of avocado straws made them a good choice.

The behaviors of the spermatozoa during refrigeration with both the avocado and bamboo straws were truly surprising, since both total and progressive motility levels were similar to the control; in the case of avocado, we could observe that it was the most stable straw in regard to maintaining total motility and good conditions over time. Nevertheless, chilling equine semen with the conventional package had great results; in fact, the results at 96 h for all our total motility values were lower than those obtained by Arruda de Oliveira with the traditional package, who obtained progressive motility results close to 80% after 72 h of refrigeration [25]. The extender used by them, Equiplus^®^, was similar to our extender, but their results were probably better than ours because they experimented with spermatozoa from ejaculate, not from the epididymis, which is more sensitive to cryopreservation; however, the use of epididymal spermatozoa is a trend because it is a good option for genetic preservation. In future, studies with ejaculated semen should be developed to validate the use of these bamboo and avocado straws as an alternative to the use of commercial plastic straws.

During cryopreservation, avocado straws showed the best total motility results at any time, even better than the control, but the quality of motility decreased over time, as shown in other seminal refrigeration studies [26,27]. At 24 h, there were no large differences with respect to bamboo straws, but there were large differences with respect to the control. Nevertheless, at 96 h, bamboo showed the poorest results and avocado still had better results, with a remarkable value of 56.27 ± 24.75. The composition of the avocado straw potentially affected the extended sperm due to its avocado seed composition, which has different polyphenol compounds, such as 3-O-caffeoylquinic acid, 3-O-p-coumaroylquinic acid, procyanidin trimer, procyanidin trimer, catechin, and epicatechin gallate [28]. Some of these compounds positively influence semen quality because of their antioxidant properties, such as catechin, which prevents the action of reactive oxygen species (ROS) present in cryopreservation protocols. Segovia [29] demonstrated that the antiradical activity of avocado seed occurred due to polyphenols (+)-catechin, (−)-epicatechin, and 3-O-caffeoylquinic acid (chlorogenic acid isomer). There have been few investigations regarding the influences of these substances on semen, and some results could provide a novel mechanism involving estrogen receptors through which low doses of epigallocatechin gallate could provide benefits in terms of sperm physiology. The detected data also showed the adverse action of high EGCG concentrations in humans, which were probably related to their pro-oxidant and antiestrogenic potential [30]. This phenomenon was confirmed in frozen goat semen [31]. It is possible that this substance was somehow present in the straws and benefitted the sperm quality.

Conversely, the results obtained with the control could be related to the plastic of the package because a majority of them used nonionic surfactants, alkylphenol ethoxylates, and hydrolysis products, such as nonylphenol tris(nonylphenyl) phosphite. Nonylphenols could cause oxidative stress by the production of ROS, such as hydrogen peroxide and superoxide anion. ROS could damage cellular components and cause cell death. In fact, the addition of nonylphenol at high concentrations (10 ppm and 20 ppm) produced very significant decreases in motility, viability, and mitochondrial activity and an increase in early apoptosis; additionally, acrosomes reacted after only 1 day of exposure [32].

However, the evaluation of total sperm motility was important, and kinetic parameters, such as curvilinear, straight line, and average path velocities, were essential components of sperm quality. Comparing our results in terms of linearity indices, we could observe that they coincided with those of Giaretta [33] (always 38.20 ± 5.70) and were higher than those obtained by Gacem [34] (always 26.99 ± 11.95). The same result occurred with the straightness index, 69.25 ± 7.13 and 36.36 ± 15.50, for Giaretta and Gacem, respectively. In both cases, these results were within the normal mean; therefore, we guaranteed that the quality of the sperm in both straws tested was reliable in terms of these kinetic parameters. However, the oscillation index was higher than that obtained by Giaretta (72.73 ± 7.53 vs. 54.21 ± 6.94) and was within the range of the values obtained by Gacem (74.22 ± 9.91). Notably, both research terms used sperm from ejaculates and the conventional package. Nevertheless, although we followed different storage procedures from the usual techniques, the values obtained were maintained as if they had been carried out under standard conditions.

Related to velocities, the results obtained with the avocado straws were better than those of the control and those of bamboo as the speeds were stable and decreased slowly. With these results, we could observe that it was possible to refrigerate the semen using avocado straws for 96 h. Other studies, although with conventional packaging, obtained good refrigeration results for 72 h with speeds similar to those obtained in our work [35]. The speeds that we observed in the control and in the bamboo straws were similar at the beginning, decreasing significantly throughout the refrigeration control process before finally leaving the results of the bamboo straws below the control value.

Since there was no bacterial growth in the bamboo and avocado straws tested, we can report that their use could be an option for reducing antibiotic use and a measure against growing antibiotic resistance due to their excessive use.

The raw material from which the straws were made could be the reason. In the case of bamboo, its antifungal capacity was associated with its chemical composition [36]. Conversely, polyphenol content in avocado seeds could protect against microorganisms and prevent lipid peroxidation due to the attack of free radicals [37]. This content was seemingly effective against bacteria. Egbuonu [15] reported that the avocado seed extract elicited antibacterial activity (mm) against *Proteus mirabilis* (23 ± 0.14), *Staphylococcus aureus* (16 ± 0.04), and *Pseudomonas aeruginosa* (15 ± 0.11); however, the reductions featured lower degrees than the standard amount with ciprofloxacin.

In seminal ejaculates, it is common to find bacterial growth due to contamination induced by the procedure used to obtain the sample itself, and the most common microorganisms are Firmicutes, Bacteroidetes, Actinobacteria, and Proteobacteria [38]. In our case, the process of working with epididymis samples and obtaining the sample was relatively controlled, making contamination unlikely. However, in the samples already processed, we could find contamination in the straws, which has been demonstrated in some studies [39].

## 5. Conclusions

In assessing the obtained motility data, avocado straws together with bamboo straws are an option for the immediate future in regard to reducing the use of plastic in sperm preservation processes for the equine species, with avocado straws improving preservation over time significantly compared to the usual plastic material used; additionally, avocado has proven to be reliable in terms of maintaining seminal motility and safe in terms of possible contamination.

## Figures and Tables

**Table 1:** Commercial brands, compositions, and dimensions of the straws used in the study.

Commercial Brand	Composition	Length (cm)	Diameter (mm)
Bamboo (Pandoo^®^)	70% bamboo + 30% vegetable starch	21	4
Avocado (Biofase^®^)	60% avocado seed biopolymer + 40% synthetic organic compounds	21	6
Herb (Waldlieb^®^)	100% herb	20	4.5–6.5
Paper (Eco Natural^®^)	100% vegetal fibers	20	6
Kraft paper (Comfy Package^®^)	100% vegetal fibers	20	6
Wheat (New Living^®^)	100% wheat	20	3.5–5
Rice (Rice Sips^®^)	70% rice + 30% cassava	21	6

**Table 2:** Wettability measurements for the different straws: inclination angle (°) and volume (µL).

	Inclination Angle (°)		Volume (µL)	
	0 s	6 s	0 s	6 s
Paper	55.8	47.3	1.8	1.2
Kraft paper	66.7	27.2	2.3	1.13
Herb	59	40.7	1.60	0.9
Rice	-	-	-	-
Wheat	60	0	1.60	-
Bamboo	60.3	60.1	3.5	3.5
Avocado	71.2	71.5	7.8	7.7

## Data Availability

The original contributions presented in this study are included in the article. Further inquiries can be directed to the corresponding author.
